# A novel AgBr/BiFeO_3_ Z-scheme heterojunction with boosted photocatalytic dye removal

**DOI:** 10.1039/d5ra08822d

**Published:** 2025-12-08

**Authors:** Shuai Fu, Yun Wen, Qiang Huang, Yonglong Song, Qi Liu, Huijie Zhu, Yanhong Wang, Tingting Feng, Shichao Liu, Zhiqun Ma

**Affiliations:** a Henan Key Laboratory of Green Building Materials Manufacturing and Intelligent Equipment, School of Intelligent Construction and Civil Engineering, Luoyang Institute of Science and Technology Luoyang 471023 PR China shuaifu16@163.com huijiezhu@lit.edu.cn; b Henan Engineering Research Center of Water Quality Safety in the Middle-lower Yellow River, Henan Green Technology Innovation Demonstration Base Luoyang 471023 PR China

## Abstract

The Z-scheme AgBr/BiFeO_3_ heterojunction was successfully constructed *via* a combined hydrothermal and *in situ* precipitation strategy. The optimized heterojunction exhibited remarkable photocatalytic activity, achieving 95.72% degradation of Lanasol Red 5B within 60 min under visible-light irradiation, which represented a 5.66-fold enhancement over pristine BiFeO_3_. Comprehensive characterization revealed that the Z-scheme heterostructure effectively facilitated charge carrier separation and modified electron transfer pathways. The experimental factor towards degradation efficiency of Lanasol Red was investigated. Furthermore, the AgBr/BiFeO_3_ heterojunction demonstrated versatile degradation capability toward various organic dyes including Lanasol Red 5B, rhodamine B, methyl orange, methyl red, and methylene blue. Trapping experiments combined with ESR analysis verified that holes (h^+^) and superoxide radicals (˙O_2_^−^) played critical roles in the photocatalytic process, which formed the basis for postulating a Z-scheme charge transfer mechanism. This work provided a feasible synthesis strategy for developing high-performance BiFeO_3_-based photocatalytic systems for environmental remediation applications.

## Abstract 2

Proposed charge transfer and photocatalytic mechanisms over the AgBr/BiFeO_3_ heterojunction.

## Graphical abstract

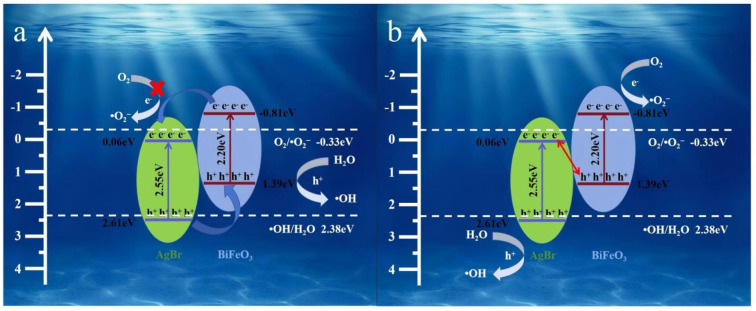

## 1. Introduction

The contamination of ecosystems by man-made organic compounds has escalated into a critical global concern, significantly endangering both environmental stability and public health.^1,2^ Lanasol Red 5B (LR5B) is a reactive dye extensively employed in textile manufacturing. Characterized by a stable chemical nature, intricate aromatic framework, and limited susceptibility to biological decomposition, LR5B resists effective elimination through traditional wastewater treatment approaches.^3,4^ In response to synthetic dye contamination, multiple remediation strategies have been explored, including microbial degradation,^5^ sorptive removal,^6^ membrane filtration,^7^ and light-driven catalytic processes.^8^ Photocatalysis, in particular, has attracted considerable interest due to its cost-effectiveness, high degradation performance, and eco-friendly characteristics.^9–13^ While physical adsorption only relocates contaminants and biological treatment is often impeded by toxic conditions, photocatalysis utilizes light energy to mineralize hazardous pollutants into harmless end products like water and carbon dioxide under ambient conditions, which positions it as a sustainable alternative for effective water treatment.^14–16^

Bismuth ferrite (BiFeO_3_), a representative multiferroic perovskite semiconductor, demonstrates coexisting ferroelectric and antiferromagnetic behavior under ambient conditions.^17–19^ These distinctive characteristics make it a promising candidate for photocatalytic applications. Additionally, its relatively small band gap allows efficient utilization of visible light, while its robust chemical stability further supports its potential in photocatalysis.^20,21^ Nevertheless, the practical deployment of BiFeO_3_ faces limitations due to certain inherent weaknesses. These include a constrained spectrum of visible-light absorption and rapid recombination of photogenerated charge carriers, which collectively lead to unsatisfactory photocatalytic performance.

To address these challenges, the formation of heterojunctions has been widely investigated as a viable approach. For example, Marouani *et al.* prepared a CaIn_2_S_4_BiFeO_3_ Z-scheme heterojunction by a simple hydrothermal method, which showed a pseudo-first-order kinetic rate constant (*k* = 0.049 min^−1^), surpassing those of pristine BiFeO_3_ (0.006 min^−1^) and CaIn_2_S_4_ (0.013 min^−1^) toward TC degradation. Electron spin resonance and radical quenching experiments confirmed that superoxide and hydroxyl radicals were the dominant reactive species facilitating TC degradation. The enhanced photocatalytic activity is attributed to the construction of the Z-scheme heterojunction, which facilitated charge separation and improved redox ability.^22^ Lin *et al.* successfully synthesized BiFeO_3_/CdS/Ti_3_C_2_ heterojunction, which exhibited exceptional piezo-photocatalytic activity, achieving 98.0% RhB degradation within 120 min (rate constant: 0.0288 min^−1^), representing a 12.68-fold enhancement over pristine BiFeO_3_. This performance enhancement originates from the enhanced light absorption and charge separation efficiency.^23^

The distinctive charge transfer mechanism of Z-scheme heterostructures makes them highly attractive for photocatalytic applications. In these systems, a selective annihilation occurs between electrons in one semiconductor and holes in the other. This process preserves the most energetic charge carriers, thereby achieving a superior combination of efficient charge separation and potent redox capability.^24,25^ For example, Samarasinghe *et al.* successfully synthesized a MoS_2_/Fe_2_O_3_/GO Z-scheme heterojunction by a ball milling and ultrasonication method. The as-synthesized optimal MFG composite with an optimal ratio of 2 : 1 : 1 exhibited remarkable photocatalytic activity towards Methylene Blue degradation, achieving a 97.90% degradation in 3 h under solar simulated irradiation and 88.2% degradation under natural sunlight. The MoS_2_/Fe_2_O_3_/GO Z-scheme heterojunction facilitated efficient charge carrier separation, extended the visible light absorption and subsequently improved the photocatalyst's dye degradation and redox capabilities.^26^ Luo *et al.* synthesized a novel SnIn4S8@ZnO Z-scheme heterostructure with a tight contact interface using a convenient two-step hydrothermal approach. The optimized heterostructure displayed the highest photodegradation efficiency toward MB (91%) after 20 min. The improvement in photocatalytic activity could be ascribed to the efficient spatial separation of photoinduced charge carriers through a Z-scheme heterojunction with an intimate contact interface.^27^

AgBr has emerged as a prominent visible-light-responsive photocatalyst, primarily due to its narrow band gap, which enables efficient absorption of a significant portion of the solar spectrum. Its exceptional photosensitivity allows for the rapid generation of electron–hole pairs upon visible-light irradiation, leading to high quantum efficiency in the initial steps of the photocatalytic process.^28,29^ However, the photogenerated electrons could readily reduce Ag^+^ to metallic Ag, causing severe photo-corrosion and rapid degradation of the catalyst structure. The instability poses a major obstacle to practical and long-term application. Consequently, significant research efforts are directed toward stabilizing AgBr, often by coupling it with other semiconductors or protective materials to facilitate efficient charge separation and prevent self-decomposition, thereby harnessing its high initial activity while mitigating its inherent limitations.^30–32^ For example, Warshagha *et al.* fabricated a highly effective and unique AgBr–NiO binary heterojunction using an effective one-pot sol–gel method that achieved 97.6% removal of RhB within 11 min, owing to the Z-scheme heterojunction.^33^ Similarly, Zhang *et al.* synthesized a novel Z-scheme heterojunction photocatalysts of AgBr/CoWO_4_/Ag *via* a simple hydrothermal-precipitation-photoreduction method. The 5AgBr/CoWO_4_/Ag exhibited the highest degradation rates, reaching 98.58% for Rhodamine B, 86.82% for tetracycline hydrochloride, and 95.60% for 2-mercaptobenzothiazole within 60 min. The enhanced photocatalytic performance could be attributed to the electron mediation by Ag nanoparticles leading to improved charge separation efficiency and the formation of Z-scheme heterojunctions between AgBr and CoWO_4_.^34^

Based on a straightforward hydrothermal-precipitation approach, this work fabricated a Z-scheme AgBr/BiFeO_3_ heterojunction. The as-prepared heterojunction was thoroughly examined by means of XRD, SEM, XPS, UV-vis DRS, and PL spectroscopy to determine its structural, morphological, optical, and electrical characteristics. Photocatalytic performance was assessed through the decomposition of five dye pollutants under visible-light irradiation. Optimal performance was observed with the 15 wt% AgBr/BiFeO_3_ heterojunction. Furthermore, the influences of several environmental parameters (dosage of catalyst, initial LR5B concentration, coexisting ions, and different aqueous matrices) on the photocatalytic process were investigated, and a plausible Z-scheme mechanism was proposed.

## 2 Experimental

### 2.1. Chemicals

All chemicals were of analytical reagent grade and used without further purification. Bismuth nitrate pentahydrate (Bi(NO_3_)_3_·5H_2_O) was obtained from Shanghai Adamas Reagent Co., Ltd. Iron(iii) nitrate nonahydrate (Fe(NO_3_)_3_·9H_2_O) and potassium bromide (KBr) were sourced from Aladdin Reagent Co., Ltd. Potassium hydroxide (KOH), silver nitrate (AgNO_3_), disodium ethylenediaminetetraacetate (EDTA-2Na), 1,4-benzoquinone (BQ), and isopropyl alcohol (IPA) were purchased from Sinopharm Chemical Reagent Co., Ltd. Deionized water was utilized throughout all experimental processes.

### 2.2. Synthesis of BiFeO_3_

The BiFeO_3_ catalyst was synthesized *via* a facile hydrothermal method adapted from literature.^35^ In a typical procedure, 3.88 g of Bi(NO_3_)_3_·5H_2_O and 3.23 g of Fe(NO_3_)_3_·9H_2_O were dissolved in 30 mL of 10 M KOH solution under continuous stirring at room temperature for 50 min, followed by 30 min of ultrasonication to form a homogeneous suspension. The resulting mixture was transferred into an 80 mL Teflon-lined stainless steel autoclave and subjected to hydrothermal treatment at 190 °C for 6 h. After natural cooling to ambient temperature, the brownish-red precipitate was collected by centrifugation, washed thoroughly, and dried at 80 °C for 12 h to obtain the final BiFeO_3_ product.

### 2.3. Synthesis of AgBr/BiFeO_3_

**Fig. 1:**
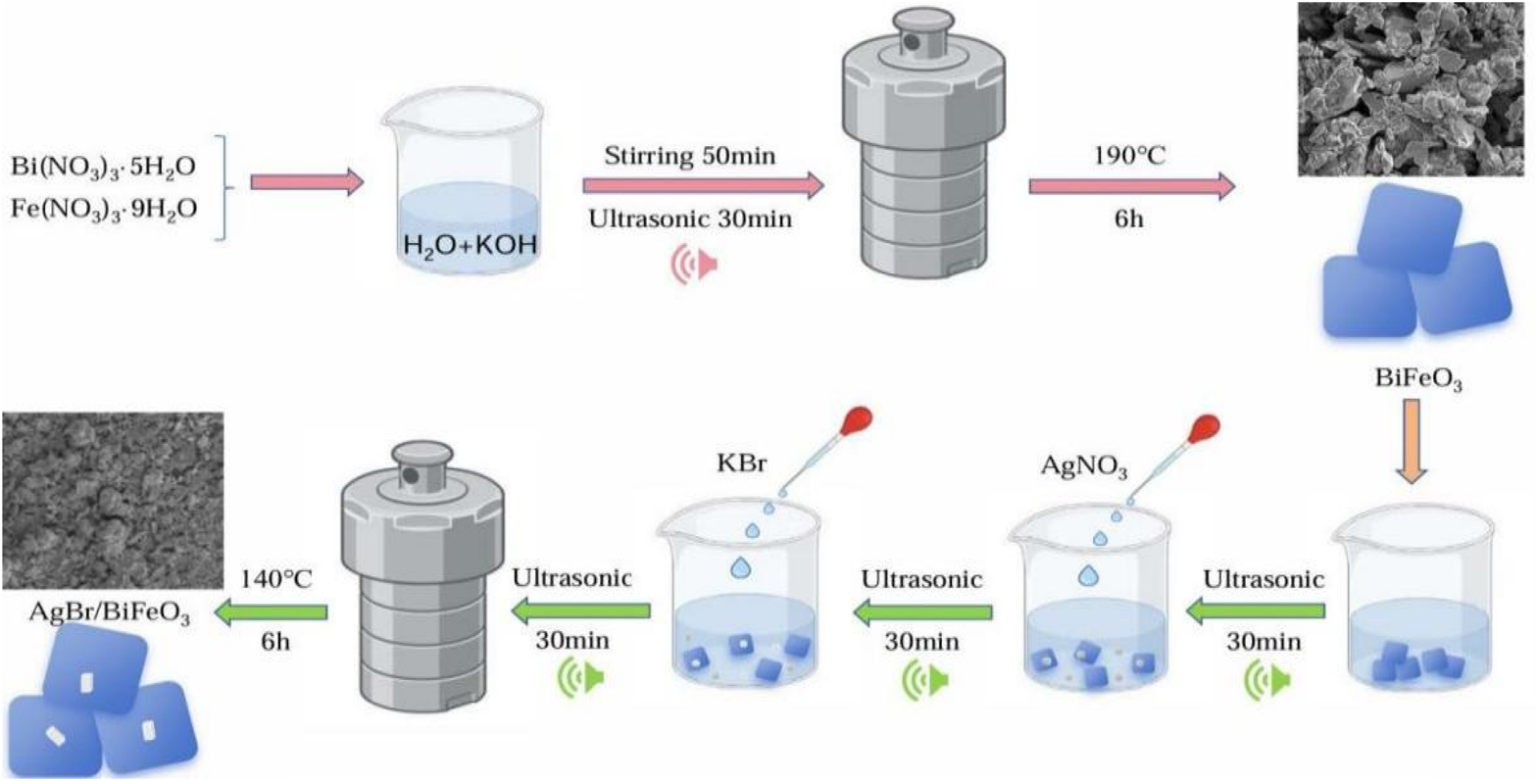
A synthesis scheme of the AgBr/BiFeO_3_ heterojunction.

The synthesis of AgBr/BiFeO_3_ heterojunction began by dispersing 0.2 g of BiFeO_3_ in 50 mL of deionized water under ultrasonication for 30 min to achieve a homogeneous suspension. Next, 4 mL of a 0.05 mol per L AgNO_3_ solution was introduced dropwise, followed by an additional 30 min of ultrasonication. Subsequently, 2 mL of 0.1 mol per L KBr was incorporated, and the mixture was subjected to further ultrasonication for 1 h. The resulting suspension was then transferred into an 80 mL autoclave and maintained at 140 °C for 6 h. After cooling naturally to ambient temperature, the final product was collected and washed three times alternately with deionized water and anhydrous ethanol, then vacuum-dried at 60 °C over 24 h. The as-synthesized AgBr/BiFeO_3_ samples with AgBr mass fractions of 10%, 15%, and 20%, were designated as AB-10, AB-15, and AB-20, respectively. For comparative analysis, pure AgBr was also synthesized using an identical procedure in the absence of BiFeO_3_. A schematic illustration of the stepwise fabrication process for the AgBr/BiFeO_3_ heterojunction was presented in Fig. 1.

### 2.4. Characterization

The crystalline structures of the synthesized samples were analyzed by X-ray diffraction (XRD) on a Bruker-D8 diffractometer equipped with Cu Kα radiation (*λ* = 0.15406 Å), with data collected over a 2*θ* range from 10° to 80°. Chemical states and surface composition were investigated using X-ray photoelectron spectroscopy (XPS) on a Thermo Escalab 250 instrument, where binding energies were referenced to the adventitious carbon C 1s peak at 284.7 eV. Sample morphology was characterized by scanning electron microscopy (SEM, SU8010). Optical absorption properties were evaluated by UV-vis spectroscopy (Perkin Elmer) across the 200–800 nm wavelength range, while photoluminescence (PL) spectra were acquired using a Pick Quant FLuo Time 3000 fluorescence spectrometer to study charge carrier behavior.

### 2.5. Evaluation of the visible-light-driven photocatalytic performance

The photodegradation performance of the as-synthesized AgBr/BiFeO_3_ heterojunction was assessed by decomposing LR5B under visible-light irradiation from a 350 W xenon lamp. In a standard procedure, 40 mL of LR5B solution (40 mg L^−1^) containing the catalyst (1 g L^−1^) was first magnetically stirred in darkness for 30 min to establish adsorption–desorption equilibrium. At designated time intervals during illumination, 4 mL aliquots were collected, centrifuged, and analyzed using a UV-2450 ultraviolet-visible spectrophotometer to determine the residual LR5B concentration. The degradation efficiency was quantified as (*C*_0_ – *C*_*t*_)/*C*_0_, where *C*_0_ represents the initial dye concentration and *C*_*t*_ corresponds to the concentration at time *t*.

## 3 Results and discussion

### 3.1. Physicochemical properties

**Fig. 2:**
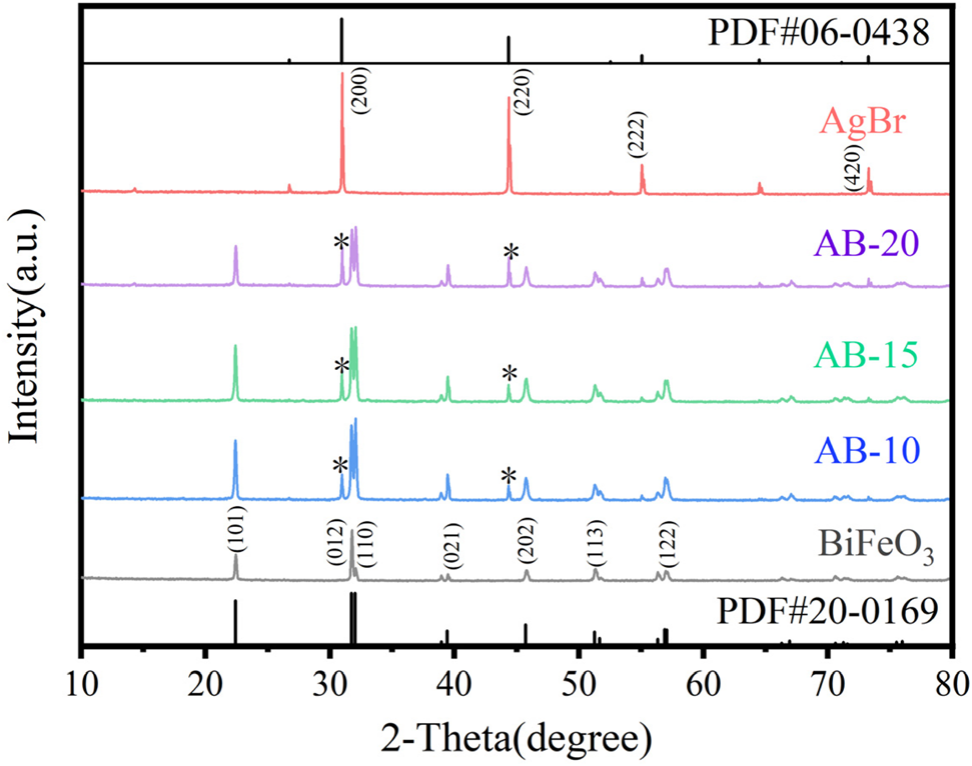
XRD patterns of BiFeO_3_, AgBr and AgBr/BiFeO_3_ with different AgBr loading amount.

The structure of AgBr and BiFeO_3_ as well as a series of AgBr/BiFeO_3_ heterojunction were studied by XRD. As shown in Fig. 2, the pure BiFeO_3_ sample exhibited distinct diffraction peaks at 2*θ* = 22.49°, 31.81°, 32.14°, 39.51°, 45.81°, 51.37°, and 57.01°, corresponding to the (101), (012), (110), (021), (202), (113), and (122) crystal planes of the BiFeO_3_ (JCPDS 20-0169), respectively, which belonged to the rhombohedral crystal system. AgBr exhibited characteristic diffraction peaks at 2*θ* = 30.94°, 44.33°, 55.04°, and 73.24°, corresponding to the (200), (220), (222), and (420) crystal planes of the AgBr (JCPDS 06-0438), respectively. The AgBr/BiFeO_3_ heterojunction showed diffraction peaks consistent with those of rhombohedral BiFeO_3_ as a whole. However, as the AgBr content gradually increased, the characteristic diffraction peak of AgBr gradually appeared, indicating the formation of AgBr/BiFeO_3_ heterojunction.

**Fig. 3:**
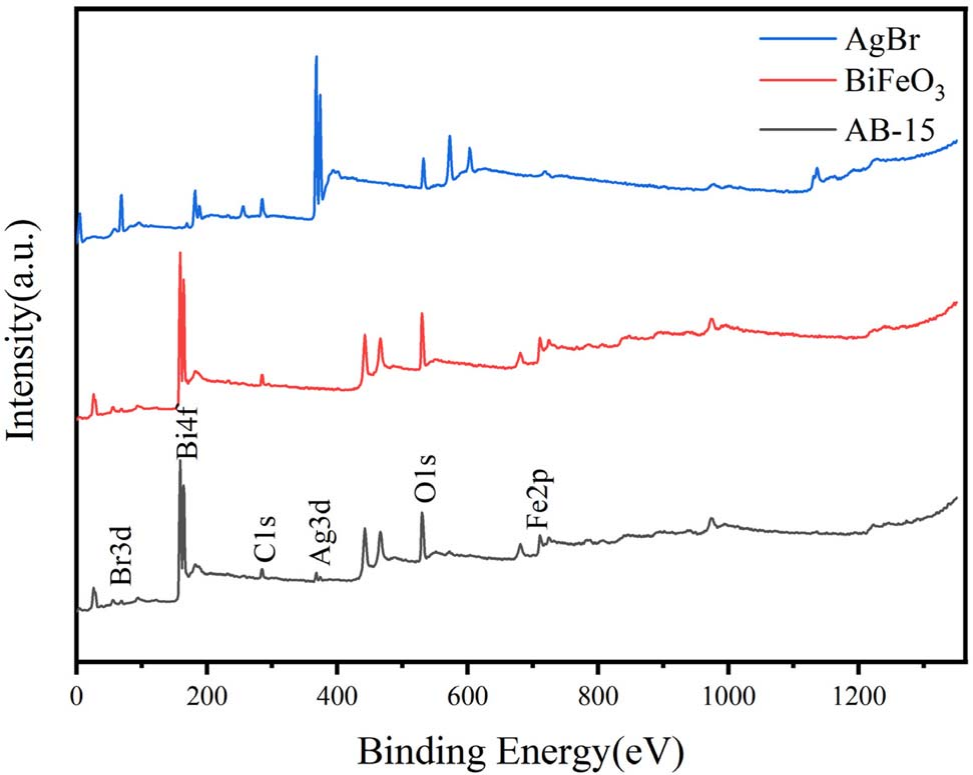
XPS spectra of BiFeO_3_, AgBr and AB-15 heterojunction.

**Fig. 4:**
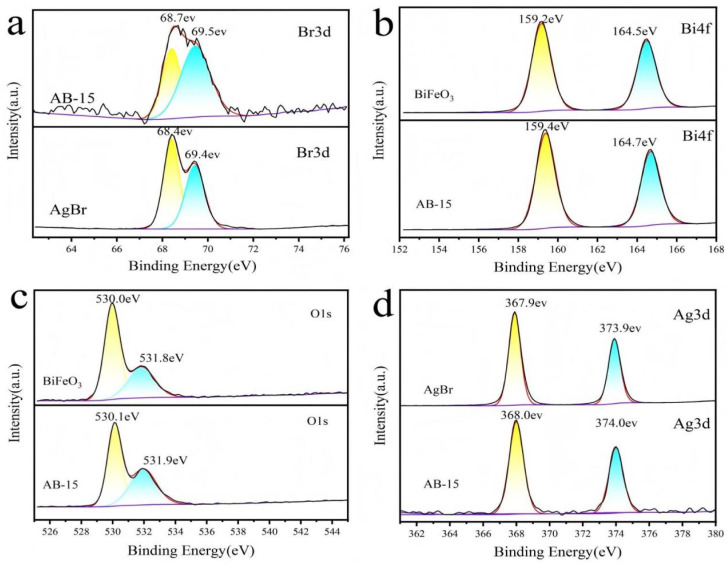
XPS spectra of BiFeO_3_, AgBr, and AB-15 heterojunction: (a) Br 3d, (b) Bi 4f, (c) O 1s, (d) Ag 3d.

X-ray photoelectron spectroscopy (XPS) characterization was systematically performed to elucidate the elemental composition and oxidation states of the synthesized BiFeO_3_, AgBr, and AB-15 heterostructure. The wide-scan spectrum of AB-15 (Fig. 3) demonstrates co-existence of constituent elements Bi, Fe, O, Ag, and Br. Deconvoluted high-resolution spectra provide precise chemical state analysis. The Br 3d core-level spectrum (Fig. 4a) exhibited spin–orbit splitting components at 68.7 eV (3d_5/2_) and 69.5 eV (3d_3/2_), characteristic of bromide anions. The Bi 4f spectrum (Fig. 4b) revealed doublet peaks at 159.4 eV (4f_7/2_) and 164.7 eV (4f_5/2_), confirming the trivalent state of bismuth.^36^ Spectral deconvolution of O 1s region (Fig. 4c) resolved two distinct contributions at 530.1 eV (metal–oxygen bonds) and 531.9 eV (surface-adsorbed oxygen species).^37^ Furthermore, the Ag 3d spectrum (Fig. 4d) displayed symmetrical peaks at 368.0 eV (3d_5/2_) and 374.0 eV (3d_3/2_), consistent with silver cations in AgBr lattice. Critically, comparative analysis showed systematic positive binding energy shifts across all elemental peaks in AB-15 relative to pristine counterparts. These shifts signified substantial interfacial electron redistribution and strengthened electronic coupling between BiFeO_3_ and AgBr phases. The modified electron cloud density provides unequivocal evidence for successful heterojunction formation with optimized electronic structure.^38^

**Fig. 5:**
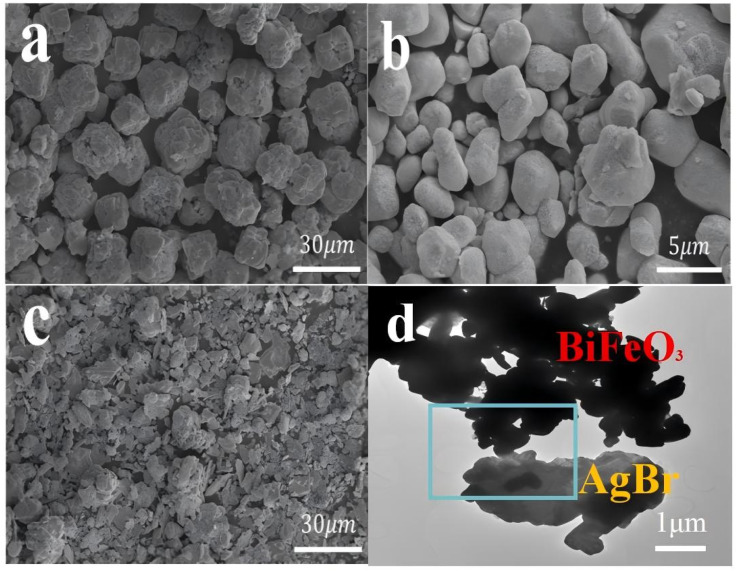
SEM images of (a) BiFeO_3_, (b) AgBr and (c) the AB-15 heterojunction, (d) TEM images of the AB-15 heterojunction.

**Fig. 6:**
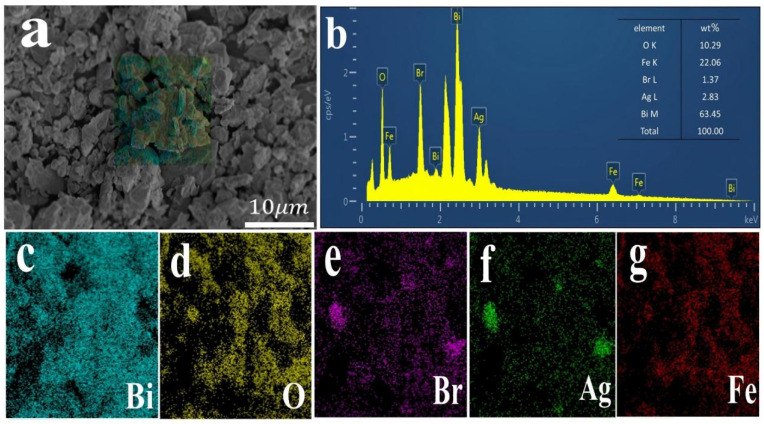
(a–g) EDS elemental mapping of the AB-15 heterostructure.

Fig. 5 showed SEM images of BiFeO_3_, AgBr and AB-15 heterojunction. It could be seen that pure BiFeO_3_ was composed of a large number of irregular polyhedra stacked together with a diameter of about 8–10 µm (Fig. 5a). As shown in Fig. 5b, pure AgBr nanoparticle exhibited a nano block structure with a diameter of around 1–5 µm. In Fig. 5c, AgBr nanoparticle adhered to the surface of BiFeO_3_, confirming the good interfacial contact between AgBr and BiFeO_3_, which was conducive to the interfacial reaction. Further structural characterization of the AB-15 heterojunction was performed using TEM. As shown in Fig. 5d, AgBr nanoparticles were intimately anchored on the surface of BiFeO_3_. In EDS spectrum, the Ag, Br, Bi, Fe, Br and O five elements were distributed on the surface of AB-15 heterojunction, which indicated that the heterojunction was composed of AgBr and BiFeO_3_, further proving the formation of AgBr/BiFeO_3_ heterojunction (Fig. 6).

**Fig. 7:**
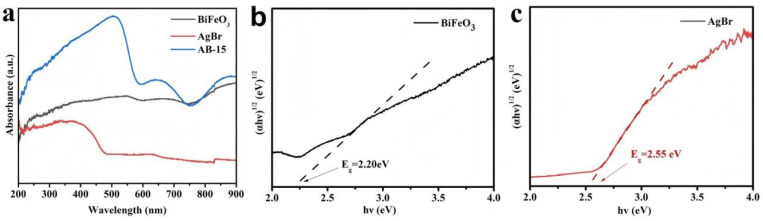
(a) UV-vis spectra, (b and c) band gap energy of BiFeO_3_ and AgBr.

**Fig. 8:**
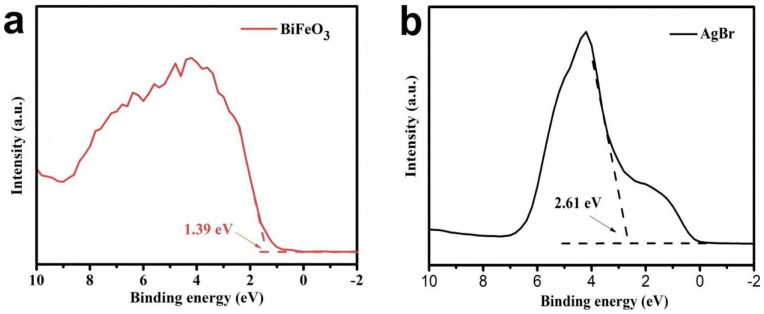
Valence-band XPS spectra of (a) BiFeO_3_ and (b) AgBr.

The UV-vis diffuse reflectance spectra of the as-synthesized photocatalyst demonstrated broad absorption across both ultraviolet and visible regions (Fig. 7a). The optical band gaps were determined using the Tauc plot method based on the equation: *αhν* = *A*(*hν* − *E*_g_)^*n*/2^, where *α*, *h*, *ν*, *A*, and *E*_g_ represent absorption coefficient, Planck's constant, light frequency, proportionality constant, and band gap energy, respectively. The derived band gap energies for BiFeO_3_ and AgBr were approximately 2.20 eV and 2.55 eV, respectively (Fig. 7b and c). Valence band X-ray photoelectron spectroscopy (VB-XPS) was employed to determine the electronic band structures. As shown in Fig. 8a and b, the valence band (VB) positions of BiFeO_3_ and AgBr were measured at 1.39 eV and 2.61 eV, respectively. Subsequently, the corresponding conduction band (CB) positions were calculated to be −0.81 eV for BiFeO_3_ and 0.06 eV for AgBr using the relationship: *E*_CB_ = *E*_VB_ − *E*_g_.^39^

### 3.2. Photocatalytic activity

**Fig. 9:**
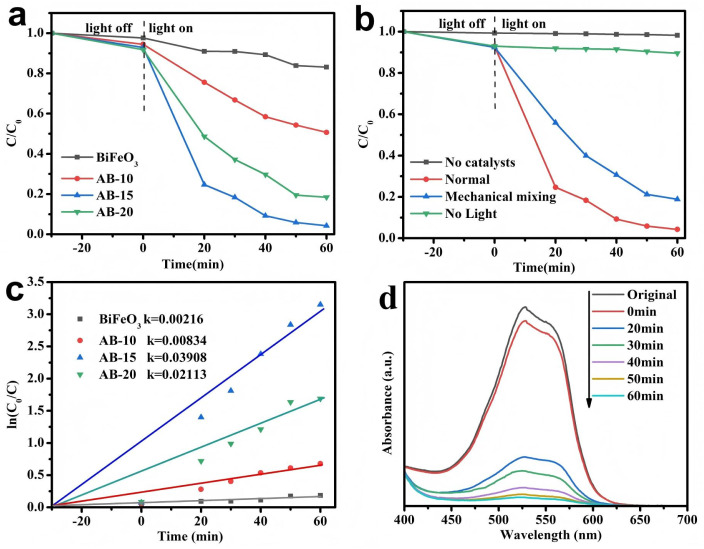
(a) Degradation efficiencies and (b) contrast experiment of LR5B using as-prepared photocatalysts (c) kinetic curves (d) time-dependent absorption spectra of LR5B degradation efficiency using AB-15 heterostructures under visible-light irradiation.

In this experiment, the dye LR5B was selected as the characteristic pollutant model to evaluate the photocatalytic performance of the as-prepared material. The experiment first conducted a 90 min dark reaction (Fig. 9b) to determine the adsorption–desorption characteristics of the composite. The removal rate of LR5B remained basically unchanged at 30 min and 90 min, indicating that the composite had reached adsorption–desorption equilibrium at 30 min. The comparative experimental results showed that LR5B hardly degraded under visible-light irradiation without the addition of photocatalyst. As shown in Fig. 9a, within a reaction time of 60 min, the degradation rate of LR5B by pure BiFeO_3_ was 16.89%. The degradation efficiency of LR5B by AgBr/BiFeO_3_ heterostructure first increased and then decreased. Among them, the photocatalytic activity of 15% AgBr/BiFeO_3_ heterostructure was the most outstanding, and the degradation rate of LR5B reached 95.72%. This indicated that AgBr played an important role in the photocatalytic degradation process, mainly because the increase of AgBr was beneficial for enhancing the light absorption ability and promoting the separation of photo-generated charge carriers. However, excessive AgBr might form electron–hole recombination centers, thereby inhibiting the photocatalytic degradation of LR5B. The photocatalytic reaction followed a quasi-first-order kinetic equation. As shown in Fig. 9c, the kinetic rate constant (*k*) of the AB-15 heterojunction was approximately 0.03908 min^−1^, which was 18.10, 4.68, and 1.84 times than that of pure BiFeO_3_, AB-10 and AB-20. Further observation of the photocatalytic degradation of LR5B by AB-15 heterojunction under simulated sunlight was conducted through real-time monitoring of UV-visible absorption spectra, as shown in Fig. 9d. The results indicated that with the extension of reaction time, the characteristic absorption peak of LR5B (529 nm) gradually weakened, confirming the gradual decrease in LR5B concentration.

**Fig. 10:**
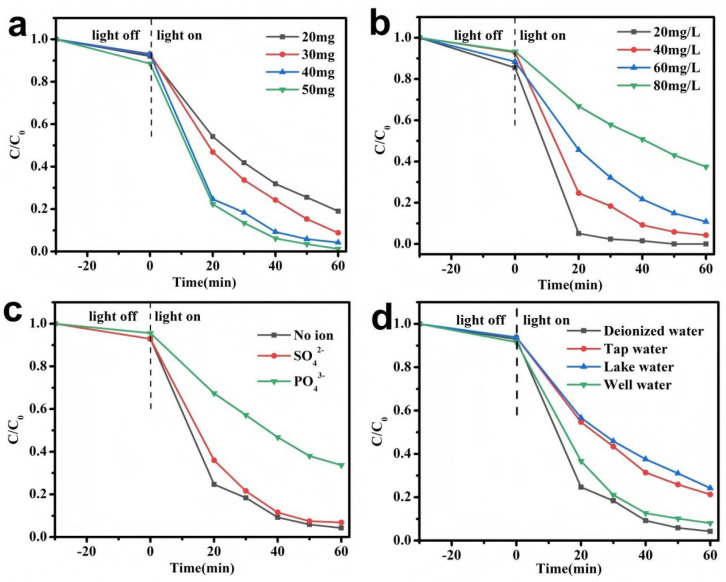
Effect of (a) different amounts of AB-15 heterojunction ([LR5B] = 40 mg L^−1^), (b) different concentration of LR5B ([AB-15] = 40 mg), (c) different inorganic salts and (d) different water resources in the presence of AB-15 under simulated solar irradiation.

The experiment investigated the effect of catalyst dosage on the degradation rate of LR5B (40 mg L^−1^), as shown in Fig. 10a. The experimental results showed that as the dosage of AB-15 gradually increased from 20 mg to 50 mg, the degradation rate of LR5B increased from 80.97% to 98.72%, which indicated that an increase in the amount of photocatalyst added could improve the efficiency of photocatalytic reactions, attributed to the fact that more catalysts promoted the formation of more active sites, thereby accelerating the degradation of LR5B. The experiment was further conducted to evaluate the degradation efficiency of the catalyst (1 g L^−1^) on LR5B at varying concentrations. As illustrated in Fig. 10b, an increase in the initial concentration of LR5B resulted in a gradual decline in the catalytic degradation efficiency. The highest photocatalytic degradation rate, achieving complete removal (100%), was observed at an initial LR5B concentration of 20 mg L^−1^. However, when the concentration was elevated to 80 mg L^−1^, the degradation rate decreased to 62.55%. This behavior could be attributed to the rapid depletion of active species generated by photocatalyst AB-15 under high LR5B concentrations. Additionally, the elevated dye concentration might impede light absorption by AB-15, thereby diminishing photocatalytic performance.

In practical wastewater systems, coexisting inorganic ions are ubiquitous and may interfere with the photocatalytic process. To simulate realistic aqueous environments, the influence of common anions (PO_4_^3−^, SO_4_^2−^) on the photodegradation of LR5B was systematically investigated. As depicted in Fig. 10c, the degradation efficiency of LR5B under 60 min irradiation was assessed in the presence of model inorganic salts (Na_3_PO_4_ or Na_2_SO_4_, 0.05 M). Notably, PO_4_^3−^ exhibited pronounced inhibition, reducing the removal efficiency to 66.31%. This suppression was likely attributable to the specific adsorption of PO_4_^3−^ onto the active sites of AB-15, where it acted as a hole scavenger, thereby passivating the photocatalytic surface.^40^ In the case of SO_4_^2−^, a moderate decrease in photocatalytic efficiency to 89.19% was observed, which could be explained by its role as a radical scavenger that consumed ˙OH radicals.

To simulate real-world conditions, the photocatalytic degradation of LR5B was evaluated in different water matrices. As shown in Fig. 10d, the degradation efficiency decreased to 78.71% in tap water, 75.74% in lake water, and 91.98% in well water. The most significant inhibition was observed in lake water, which could be attributed to its complex composition of inorganic ions and organic matter that likely scavenge active species and interfere with the photocatalytic process.

**Fig. 11:**
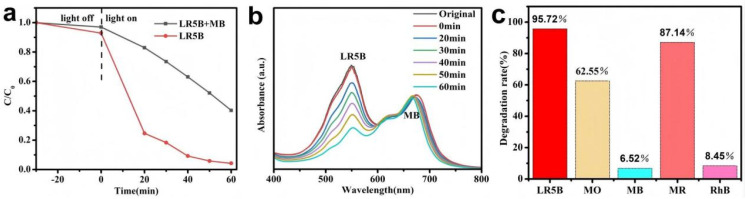
(a) Degradation efficiencies and (b) time-dependent absorption spectra of LR5B and MB, (c) degradation efficiencies of LR5B, MO, RhB, MB, MR.

To simulate complex wastewater conditions, the photocatalytic performance of the AgBr/BiFeO_3_ heterojunction was evaluated in a multi-pollutant system. As shown in Fig. 11(a and b), the degradation of LR5B was significantly suppressed in the coexistence of LR5B and MB, achieving a removal rate of only 59.69%, which could be attributed to competitive interactions between organic pollutants and active species for reactive sites and oxidative capacity. The photocatalytic performance of the AgBr/BiFeO_3_ heterojunction was further evaluated against diverse organic pollutants. As depicted in Fig. 11(c), after 60 min of reaction, the removal rates of LR5B and MR reached 95.72% and 87.14%, respectively, demonstrating the material's effective mineralization capability toward these pollutants. In contrast, significantly lower degradation efficiencies were observed for RhB and MB, with only 8.45% and 6.52% removal, suggesting their higher structural stability or more complex degradation pathways. Such pollutant-dependent performance variations could be attributed to differences in molecular structure, which influenced both the adsorption affinity to the photocatalyst surface and subsequent photocatalytic reaction kinetics.

**Fig. 12:**
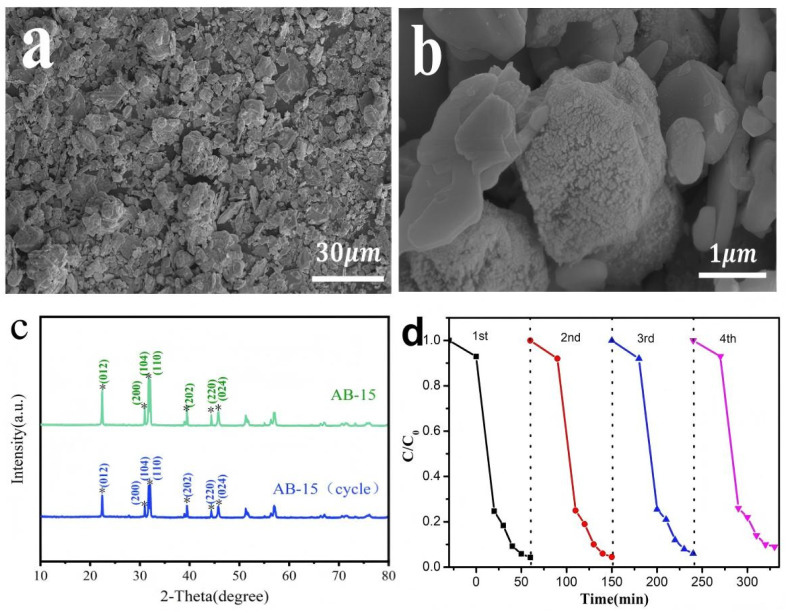
(a and b) SEM images, (c) XRD patterns of used AB-15 and fresh AB-15, (d) cycling runs for photocatalytic degradation of LR5B in the presence of AB-15.

The cycling stability and reusability of the as-prepared samples were evaluated through consecutive photocatalytic experiments. As depicted in Fig. 12(d), the AB-15 heterojunction exhibited a slight decrease in degradation efficiency for LR5B from 95.72% to 91.15% over four successive cycles. This minimal loss of activity demonstrated the robust stability and excellent reusability of the heterojunction photocatalyst. As shown in Fig. 12(a and b), no significant morphological changes were observed in the spent catalyst, though a slightly rougher surface texture was noted, possibly due to the accumulation of incompletely degraded pollutant residues. Furthermore, XRD patterns in Fig. 12(c) revealed that the crystal structure of the heterojunction remained largely unchanged after repeated use, confirming its high structural stability under photocatalytic conditions.

### 3.3. Photocatalytic mechanism

**Fig. 13:**
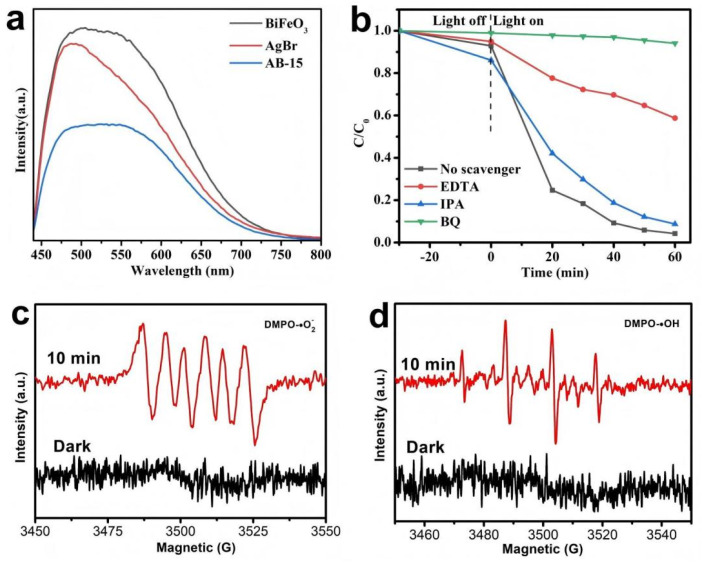
(a) PL spectra of the as-prepared samples; (b) capture tests of active species during photodegradation of LR5B by AB-15; (c) DMPO-˙OH, (d) DMPO-˙O_2_^−^ adducts on AB-15.

Steady-state photoluminescence (PL) spectroscopy was employed to investigate the recombination behavior of photogenerated electron–hole pairs in the samples. A lower PL intensity generally indicated a suppressed charge carrier recombination rate. The excitation wavelength used for PL measurements was specified as 325 nm in the experimental section. As shown in Fig. 13(a), the AB-15 heterojunction exhibited a significantly reduced emission peak intensity compared to pure AgBr and BiFeO_3_. This observation suggested that the interfacial interaction within the heterojunction effectively facilitated the separation and migration of photogenerated charge carriers, thereby minimizing radiative recombination.

In order to further understand the photocatalytic mechanism of AgBr/BiFeO_3_ heterojunction, three different capture agents were introduced in the experiment: hole (h^+^) was captured by ethylenediaminetetraacetic acid disodium (EDTA-2Na, 2 mM), superoxide radical (˙O_2_^−^) was captured by *p*-benzoquinone (BQ, 2 mM), and hydroxyl radical (˙OH) was captured by isopropanol (IPA, 5 mM), so as to explore the active substances that play a major role in the photocatalytic reaction of AgBr/BiFeO_3_ heterojunction. The experimental results (Fig. 13(b)) showed that the degradation rate of LR5B decreased sharply after the addition of BQ, which was only 5.86%. This result indicated that BQ effectively captured superoxide radicals (˙O_2_^−^), thereby significantly inhibiting the degradation of LR5B. After the addition of EDTA-2Na, the degradation rate of LR5B also decreased significantly to 41.27%, indicating that EDTA-2Na might effectively capture the holes generated during the photocatalytic process. In contrast, when the capture agent IPA was added, the degradation rate of LR5B was 91.27%, which was slightly lower than 95.72% under normal conditions. The capture experiments showed that h^+^ and ˙O_2_^−^ were the main active species, and ˙OH played an auxiliary role. In addition, electron spin resonance (ESR) spectroscopy was further employed to verify the generation of superoxide (˙O_2_^−^) and hydroxyl (˙OH) radicals in the AB-15 heterojunction system. As depicted in Fig. 13(c and d), no characteristic signals of either radical species were detected in the dark. In contrast, under visible light illumination, distinct signature peaks corresponding to the DMPO-˙OH and DMPO-˙O_2_^−^ adducts were observed. These ESR findings were in strong agreement with the radical trapping experiments, confirming the photoinduced generation of reactive oxygen species.

**Fig. 14:**
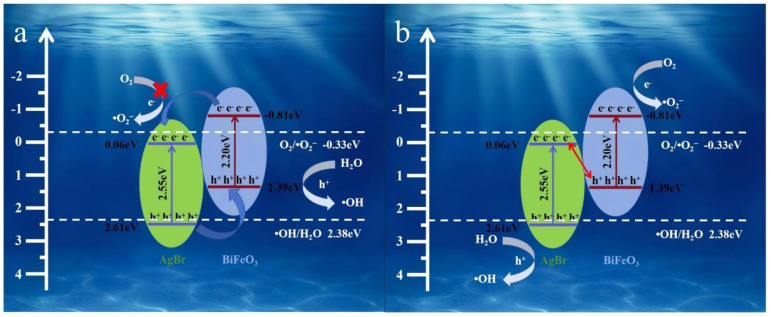
Proposed charge transfer and photocatalytic mechanisms over the AgBr/BiFeO_3_ heterojunction.

Based on a multifaceted analysis integrating radical trapping experiments, ESR spectroscopy, and XPS valence band analysis, two plausible charge transfer mechanisms for the AgBr/BiFeO_3_ heterojunction were systematically proposed and evaluated. In a conventional Type-II heterojunction model (Fig. 14a), photogenerated electrons from the conduction band (CB) of BiFeO_3_ would migrate to the CB of AgBr, while holes from the valence band (VB) of AgBr would transfer to the VB of BiFeO_3_. However, the CB potential of AgBr was determined to be +0.06 eV, which was more positive than the standard redox potential of O_2_/˙O_2_^−^ (−0.33 eV *vs.* NHE). Consequently, electrons accumulated in the CB of AgBr were thermodynamically incapable of reducing O_2_ to generate ˙O_2_^−^, which contradicted the radical trapping and ESR results. This thermodynamic inconsistency effectively ruled out the Type-II pathway as a viable mechanism. Alternatively, a direct Z-scheme mechanism was proposed, as illustrated in Fig. 14b. The establishment of this charge transfer pathway could be rationalized by considering the band bending and Fermi level alignment at the heterojunction interface. Upon contact between AgBr and BiFeO_3_, the difference in their original Fermi levels drived electron flow from BiFeO_3_ to AgBr until Fermi level equilibration was achieved. This process created a built-in electric field directed from BiFeO_3_ to AgBr, resulting in upward band bending in AgBr and downward band bending in BiFeO_3_ at the interface. Under visible-light irradiation, both semiconductors generated electron–hole pairs. The photogenerated electrons in the CB of AgBr and holes in the VB of BiFeO_3_ were subsequently driven by the built-in electric field to recombine selectively at the interface through the Z-scheme pathway. This unique recombination mechanism preserved the most reductive electrons in the CB of BiFeO_3_ and the most oxidative holes in the VB of AgBr, thereby synergistically enhancing both redox capability and charge separation efficiency. Specifically, the VB potential of AgBr was calculated as +2.61 eV, which was sufficiently positive to oxidize H_2_O to generate ˙OH (*E*(˙OH/H_2_O) = +2.38 eV *vs.* NHE). Simultaneously, the CB potential of BiFeO_3_ (−0.81 eV) was sufficiently negative to reduce O_2_ to ˙O_2_^−^. These favorable band alignments, coupled with the interfacial band bending that facilitated the Z-scheme recombination, provided compelling thermodynamic and kinetic support for the proposed mechanism, which was fully consistent with the experimental detection of both ˙OH and ˙O_2_^−^ active species.

## 4. Conclusions

A novel Z-scheme AgBr/BiFeO_3_ heterojunction photocatalyst was successfully synthesized *via* a combined hydrothermal-precipitation approach. Photocatalytic evaluation revealed that the AB-15 heterojunction exhibited the highest activity among the synthesized samples, achieving 95.72% degradation of LR5B within 60 min under visible-light irradiation, which was 5.66 times higher than that of pristine BiFeO_3_. Environmental factor studies demonstrated that high initial LR5B concentration, low catalyst loading, and the presence of phosphate anions (PO_4_^3−^) significantly suppressed the photocatalytic efficiency. The optimized catalyst also displayed notable degradation performance toward multiple dyes, with removal rates of 95.72% for LR5B, 87.14% for MR, and 62.55% for MO. Radical trapping experiments and electron spin resonance spectroscopy confirmed that holes and superoxide radicals serve as the dominant active species. A Z-scheme charge transfer mechanism was conclusively proposed based on band structure analysis, ESR results, and scavenger experiments, which accounted for the enhanced carrier separation and redox capability. The AgBr/BiFeO_3_ heterojunction represented a promising high-activity photocatalytic system for dye wastewater treatment, which offered a green and efficient strategy for addressing aqueous organic pollutants, advancing the practical application of photocatalysis in environmental remediation.

## Author contributions

Shuai Fu: conceptualization, methodology, software, investigation, formal analysis, writing – original draft, writing – review & editing. Yun Wen: software, resources. Qiang Huang: conceptualization, funding acquisition. Yonglong Song: formal analysis. Qi Liu: methodology, software, funding acquisition. Huijie Zhu: investigation, formal analysis. Yanhong Wang: investigation, data curation. Tingting Feng: investigation, software. Shichao Liu: investigation. Zhiqun Ma: investigation.

## Conflicts of interest

The authors declare that they have no known competing financial interests or personal relationships that could have appeared to influence the work reported in this paper.

## Data Availability

All data, models, and code generated or used during the study appear in the submitted article.

## References

[cit1] Caba L. M., Bodelón G., Montecelo Y. N., Miguel A. C. (2021). Sunlight-sensitive plasmonic nanostructured composites as photocatalytic coating with antibacterial properties. Adv. Funct. Mater..

[cit2] Li R., Zhang H., Hou Y., Gao L., Chu D., Zhang M. (2025). Metallacage-crosslinked free-standing supramolecular networks *via* photo-induced copolymerization for photocatalytic water decontamination. Nat. Commun..

[cit3] Narita Y., Nishi K., Matsuyama T., Ida J. (2024). Reusable isotype heterojunction g-C_3_N_4_/alginate hydrogel spheres for photocatalytic wastewater treatment. RSC Adv..

[cit4] Yoo J., Lee J., Kim J. (2024). A floating photocatalytic fabric integrated with a AgI/UiO-66-NH_2_ heterojunction as a facile strategy for wastewater treatment. RSC Adv..

[cit5] Zhao G., Li E., Li J., Xu M., Huang Q., Rong X. (2018). Effects of interfaces of goethite and humic acid-goethite complex on microbial degradation of methyl parathion. Front. Microbiol..

[cit6] Hamid A., Wilson A. E., Torbert H. A., Wang D. (2023). Sorptive removal of phosphorus by flue gas desulfurization gypsum in batch and column systems. Chemosphere.

[cit7] Wang Q., Tang X., Liang H., Cheng W., Li G., Zhang Q., Chen J., Chen K., Wang J. (2022). Effects of filtration mode on the performance of gravity-driven membrane (GDM) filtration: cross-flow filtration and dead-end filtration. Water.

[cit8] Lindner A., Lesniewicz A., Kolman A., Larowska-Zarych D., Marciniak B., Andralojc A. L. (2024). When porphyrins meet 2D materials: spectroscopic and photocatalytic properties. J. Mater. Chem. C.

[cit9] Li X., Wu X., Liu S., Li Y., Fan J., Lv K. (2020). Effects of fluorine on photocatalysis. Chin. J. Catal..

[cit10] Zhou H., Wang H., Yue C., He L., Li H., Zhang H., Yang S., Ma T. (2024). Photocatalytic degradation by TiO_2_-conjugated/coordination polymer heterojunction: Preparation, mechanisms, and prospects. Appl. Catal., B.

[cit11] Fu S., Liu X., Yan Y., Li L., Liu H., Zhao F., Zhou J. (2019). Few-layer WS_2_ modified BiOBr nanosheets with enhanced broad-spectrum photocatalytic activity towards various pollutants removal. Sci. Total Environ..

[cit12] Lu J., Bie J., Fu S., Wu J., Huang Q., He P., Yang Z., Zhang X., Zhu H., Deng P. (2022). Construction of a novel two-dimensional AgBiO_3_/BiOBr step-scheme heterojunction for enhanced broad-spectrum photocatalytic performance. RSC Adv..

[cit13] Zhu A., Ali S., Wang Z., Xu Y., Lin R., Jiao T., Qin O., Chen Q. (2023). ZnO@ Ag-functionalized paper-based microarray chip for SERS detection of bacteria and antibacterial and photocatalytic inactivation. Anal. Chem..

[cit14] Fu S., Yuan W., Yan Y., Liu H., Shi X., Zhao F., Zhou J. (2019). Highly efficient visible-light photoactivity of Z-scheme MoS_2_/Ag_2_CO_3_ photocatalysts for organic pollutants degradation and bacterial inactivation. J. Environ. Manage..

[cit15] Fu S., Yuan W., Liu X., Yan Y., Liu H., Li L., Zhao F., Zhou J. (2020). A novel 0D/2D WS_2_/BiOBr heterostructure with rich oxygen vacancies for enhanced broad-spectrum photocatalytic performance. J. Colloid Interface Sci..

[cit16] Qian Y., Zhang F., Pang H. (2021). A review of MOFs and their composites-based photocatalysts: synthesis and applications. Adv. Funct. Mater..

[cit17] Di L., Yang H., Xian T., Liu X., Chen X. (2019). Photocatalytic and photo-fenton catalytic degradation activities of Z-Scheme Ag_2_S/BiFeO_3_ heterojunction composites under visible-light irradiation. Nanomaterials.

[cit18] Mersa L., mohammadi A. S., Samarghandi M. R., Khazaei M., Asgari G. (2025). Photocatalytic degradation of amoxicillin in aqueous solutions using rGO/BiFeO_3_ nanocomposites in the presence of LED light irradiation. Sci. Rep..

[cit19] Zou C., Liu S., Shen Z., Zhang Y., Jiang N. (2017). Efficient removal of ammonia with a novel graphene-supported BiFeO_3_ as a reusable photocatalyst under visible light. Chin. J. Catal..

[cit20] Ding J., Li H., Xi G., Tu J., Tian J., Zhang L. (2023). Bandgap engineering strategy through chemical strain and oxygen vacancies in super-tetragonal BiFeO_3_ epitaxial films. Inorg. Chem. Front..

[cit21] Mani A. D., Li J., Wang Z., Zhou J., Xiang H., Zhao J., Deng L., Yang H., Yao L. (2022). Coupling of piezocatalysis and photocatalysis for efficient degradation of methylene blue by Bi_0.9_Gd_0.07_La_0.03_FeO_3_ nanotubes. J. Adv. Ceram..

[cit22] Marouani I., Hassan W. H., Soliman N. F., Singh P. K., Khan M. I., Mahariq I., Atamurotov F., Abdujabbarov A., Diab M. A. (2025). Enhanced wastewater purification and photocatalytic green energy production *via* a novel CaIn_2_S_4_-coupled BiFeO_3_ nanocomposite: Characterization and mechanistic insights. J. Water Proc. Eng..

[cit23] Lin J., Li H., Shao J., Wang D., Xu Q. (2025). Optimization of BiFeO_3_/CdS composite structure with monolayer Ti_3_C_2_ cocatalyst for enhanced piezo-photocatalytic removing RhB. J. Mol. Struct..

[cit24] Mehrdadian E., Sheibani S., Ataie A. (2024). Outstanding photocatalytic activity of a mechano-thermally synthesized Z-scheme BiFeO_3_-Fe_2_O_3_ heterostructure. J. Alloys Compd..

[cit25] Dai W., Mu J., Chen Z., Zhang J., Pei X., Luo W., Ni B. (2022). Design of few-layer carbon nitride/BiFeO_3_ composites for efficient organic pollutant photodegradation. Environ. Res..

[cit26] Samarasinghe L. V., Muthukumaran S., Baskaran K. (2025). Magnetically recoverable MoS_2_/Fe_2_O_3_/graphene oxide ternary Z-scheme heterostructure photocatalyst for wastewater contaminant removal: Mechanism and performance. J. Environ. Chem. Eng..

[cit27] Luo Q., Sun C., Zhao J., Cai Q., Yao S. (2023). Highly efficient SnIn_4_S_8_@ZnO Z-Scheme heterojunction photocatalyst for methylene blue photodegradation. Materials.

[cit28] Ma J., Xu L., Yin Z., Li Z., Song Z., Qiu J., Li Y. (2025). Boosting charge transfer of BiOBr/AgBr S-scheme heterojunctions *via* interface Br atom co-sharing for enhanced visible-light photocatalytic activity. Green Energy Environ..

[cit29] Sun W., Ahmed T., Elbouazzaoui K., Edvinsson T., Zheng Y., Zhu J. (2024). Facile fabrication of AgBr/HCCN hybrids with Z-scheme heterojunction for efficient photocatalytic hydrogen evolution. Appl. Surf. Sci..

[cit30] Wang C., Li X., Ren Y., Jiao H., Wang F. R., Tang J. (2023). Synergy of Ag and AgBr in a pressurized flow reactor for selective photocatalytic oxidative coupling of methane. ACS Catal..

[cit31] Xue X., Chen X., Zhang Z., Fan G., Ma T. (2023). Improved ionic organic pollutant degradation under visible light by Ag SPR-promoted phosphorus-doped g-C_3_N_4_/AgBr/Bi_2_WO_6_ with excellent charge transfer capacity and high surface area. J. Alloys Compd..

[cit32] Zhang J., Zhu Z., Jiang J., Li H. (2020). Synthesis of novel ternary dual Z-scheme AgBr/LaNiO_3_/g-C_3_N_4_ composite with boosted visible-light photodegradation of norfloxacin. Molecules.

[cit33] Warshagha M. Z. A., Rasool Z., Athar M. S., Muneer M., Altass H. M., Felemban R., Khder A. S., Ahmed S. A. (2025). Development of a crystalline n-AgBr/p-NiO binary heterojunction for photocatalytic degradation of organic contaminants with accompanying mineralization, adsorption, and antimicrobial studies. Nanoscale Adv..

[cit34] Zhang J., Wei J., Li J., Xiahou M., Sun Z., Cao A., feng Y. Y., Chen G., He Y. (2024). Rational design and construction of direct Z-scheme ternary heterojunction photocatalyst AgBr/CoWO_4_/Ag for efficient environmental remediation. Environ. Res..

[cit35] Su P., Zhang D., Yao X., Liang T., Yang N., Zhang D., Pu X., Liu J., Cai P., Li Z. (2024). Enhanced piezo-photocatalytic performance in ZnIn_2_S_4_/BiFeO_3_ heterojunction stimulated by solar and mechanical energy for efficient hydrogen evolution. J. Colloid Interface Sci..

[cit36] Liu G., Lin Y., Li S., Shi C., Zhang D. (2022). Mechanism and efficiency of photocatalytic triclosan degradation by TiO_2_/BiFeO_3_ nanomaterials. Water Sci. Technol..

[cit37] Kumar A., Sharma K. S., Sharma G., Al-Muhtaseb A., Naushad M., Ghfar A. A., Stadler F. J. (2019). Wide spectral degradation of norfloxacin by Ag@BiPO_4_/BiOBr/BiFeO_3_ nano-assembly: Elucidating the photocatalytic mechanism under different light sources. J. Hazard. Mater..

[cit38] Fu S., Zhu H., Huang Q., Liu X., Zhang X., Zhou J. (2021). Construction of hierarchical CuBi_2_O_4_/Bi/BiOBr ternary heterojunction with Z-scheme mechanism for enhanced broad-spectrum photocatalytic activity. J. Alloys Compd..

[cit39] Li L., Cai J., Yan Y., Zhao F., Zhou J. (2019). Flower-like direct Z-scheme WS_2_/Bi_2_O_2_CO_3_ photocatalyst with enhanced photocatalytic activity. J. Alloys Compd..

[cit40] Fu S., Du Y., Bie J., Huang Z., Hu H., Huang Q., Zhu H., Yuan W., Li L., Liu B. (2023). Facile fabrication of Z-scheme Ag_2_WO_4_/BiOBr heterostructure with oxygen vacancies for improved visible-light photocatalytic performance. J. Sci. Adv. Mater. Devices.

